# Salivary apelin hormone response and dysfunctional attitudes in adolescents in Türkiye: a relational screening model

**DOI:** 10.1186/s40359-024-01551-w

**Published:** 2024-02-09

**Authors:** Zila Özlem Kirbaş, Bülent Bayraktar, Elif Odabaşi Aktaş

**Affiliations:** 1 Faculty of Health Sciences, Department of Nursing, Bayburt University, Bayburt, Türkiye; 2 Faculty of Health Sciences, Department of Physiotherapy and Rehabilitation, Bayburt University, Bayburt, Türkiye; 3 Faculty of Health Sciences, Department of Midwifery Bayburt, Bayburt University, Bayburt, Türkiye

**Keywords:** Dysfunctional attitudes, Adolescent, Apelin, Nursing, Pediatrics nursing

## Abstract

**Background:**

Adolescence is the period in which physical and emotional changes occur through hormones, the individual acquires gender characteristics and prepares for the adult role psychologically and physically. Dysfunctional attitudes are beliefs and attitudes that can lead to depression by causing negative thoughts about oneself, others, and the future.Dysfunctional attitudes negatively affect children’s mental health. Hormones have a significant impact on human behavior and cognitive functions. However, little is known about the role and influence of hormones on dysfunctional attitudes. Apelin is a hormone responsible for controlling emotions by regulating emotional behavior. The level of dysfunctional attitudes is one of the important issues in nursing practice in terms of protecting and improving children’s mental health. However, little is known about the role and impact of hormones on dysfunctional attitudes.This study aimed to examine adolescents’ dysfunctional attitudes and salivary apelin hormone levels in terms of sociodemographic variables.

**Methods:**

The study was conducted in a relational screening model with 151 adolescents aged 9–14 years who were reported to be clinically healthy in Türkiye. Apelin hormone levels were analyzed by ELISA technique in the saliva samples of the participants. In the evaluation of dysfunctional attitudes, the relationship between the score obtained from the dysfunctional attitude scale and salivary hormone levels was evaluated.

**Results:**

In the study, a negative, strong and statistically significant correlation was found between the average salivary apelin hormone level and dysfunctional attitudes of adolescents (*p* =.000). Mean salivary hormone levels of apelin in adolescent girls and boys were 0.696 (SD 0.052) ng/ml, respectively; while 0.671 (SD 0.047) ng/ml was determined (*p* =.002), dysfunctional attitudes scale scores were 52.95 (SD 14.43); it was determined as 59.04 (SD 14.22) (*p* =.006). On the other hand, the highest average salivary apelin hormone level (*p* =.038). and the lowest level of dysfunctional attitudes were determined in adolescent girls aged 13–14 years (*p* =.028).

**Conclusions:**

In our study, we found that while the salivary apelin hormone levels of adolescents decreased, their dysfunctional attitudes increased. We found that adolescents’ dysfunctional attitudes decreased with age. In contrast, apelin hormone levels increased with age.

## Highlights

Our study revealed the relationship between the hormone apelin and dysfunctional attitudes in adolescents.

The level of dysfunctional attitudes that play a role in the development of depression is lower in female adolescents than in male adolescents.

Apelin level recommended to be used in the treatment of depression is higher in female adolescents than male adolescents.

Apelin levels increase with age in adolescents, and dysfunctional attitude levels decrease.

## Background

Adolescence is a transitional period between childhood and adulthood, as well as emotional and hormonal changes, and it is the period in which the individual’s gender characteristics are acquired and the individual is psychologically and physically prepared for the adult role. The period of entering adolescence, girls 8–13 years; 9–14 years are reported for boys [1]. Dysfunctional attitudes are cognitions that consist of non-positive beliefs formed by the individual as a result of his relationship with the outside world and his environment and that negatively affect the individual’s life [2]. Beck [3], who was the first to discuss the concept of dysfunctional attitudes, noticed that his patients had common negative cognitive thoughts during his studies in the treatment of depression and used the cognitive therapy approach in the treatment of this situation. Cognitive therapy is based on the view that the individual’s dysfunctional attitudes should be replaced with positive, healthy functional attitudes, and focuses on increasing the individual’s positive cognitions during the therapy process [4]. Dysfunctional attitudes are very strong attitudes whose foundations are laid in childhood and continue to develop throughout life along with cognitive development from childhood [5, 6]. These hard-to-change attitudes can deeply affect an individual’s emotions and behavior. Dysfunctional attitudes; It contains negative expressions, is unrealistic, rigid and overgeneralized [7]. It can also be assumed that having dysfunctional beliefs about oneself and others may have an impact on one’s social skills and relationships with others [8]. People’s need to get approval and be liked by those around them and acting perfectionist can be given as examples of dysfunctional attitudes [2, 9]. These attitudes prevent individuals from achieving their goals. The person thinks that he cannot be successful because he feels inadequate, constantly belittles himself and becomes unhappy [2, 10]. Dysfunctional attitudes play a role in the emergence of depression during adolescence [6]. It is important to detect dysfunctional attitudes in childhood, the foundations of which are laid in childhood and continue throughout life with cognitive development [5, 6].

Hormones, especially adrenal androgens, gonadocorticoids and gonadal steroids, have a significant effect on mood lability, mood intensity, moods and behaviors during adolescence [11]. Apelin is a hormone secreted from adipose tissue and known as the ligand of the G-protein-coupled receptor APJ. There are various isoforms of the hormone apelin such as apelin-12,13,17,36 [12]. It has a role in many physiological processes such as stress response, cardiovascular functions, regulation of blood pressure, angiogenesis, thermoregulation and energy metabolism regulation On the other hand, Apelin has a role in the regulation of emotional behavior by controlling emotional behavior depending on age and metabolic status [13, 14]. Stress is a common risk factor for mood disorders such as depression. Depressive disorders may be due to a negative life event, or due to serotonin, noradrenaline, or hormonal anomalies, that is, due to biological characteristics, they may be prone to depressive disorders and develop clinical depression. Dysfunctional attitudes are a condition seen in individuals experiencing depression [15, 16]. Stress causes downregulation of various cell signaling pathways such as phosphatidylinositol 3-kinase (PI3K)/Akt (protein kinase B), extracellular signal-regulated kinase (ERK), and Mammalian target of rapamycin (mTOR). Antidepressant treatments reverse depressive-like behaviors by activating these signaling pathways [17]. Apelin activates PI3K/Akt, ERK1/2 and mTOR signals and plays an important role in regulating the stress-related response of the apelinergic system by weakening N-methyl-D-aspartate (NMDA) receptor activity [18, 19]. Apelin is a relatively newly discovered neuropeptide that is the endogenous ligand of the G-protein coupled (APJ) receptor and acts by binding to APJ and regulates CNS (central nervous system) functions [20]. Apelin and APJ have a widespread but selective expression in the CNS. The localization of the limbic system in the structures that integrate many behavioral responses, especially stress and anxiety (amygdala, hypothalamus, dentate gyrus structures, as well as extra hypothalamic structures, especially the cerebroventricular system), plays a role in the regulation of behavior by increasing the level of apelin [21].

Nurses need to help individuals in childhood and adolescence, when hormonal and emotional changes occur, develop skills to effectively cope with stress and negative emotional states. In addition, nurses can provide counseling to children and adolescents not only to protect their mental and physical health but also to optimize their relationships with the environment. Although the results are limited, it is important to evaluate the effectiveness of apelin hormone levels, which are reported to have a role in regulating emotional behaviors in children, as a critical biomarker and to evaluate dysfunctional attitudes in terms of cognitive interventions against the negative effects of cognitive disorders. In the literature review, no study was found that examined dysfunctional attitudes and salivary apelin hormone levels in adolescents together.In addition to being the first in this sense, this study also serves as a reference. In this context, this study aimed to examine adolescents’ dysfunctional attitudes and salivary apelin levels in terms of sociodemographic variables.

## Methods

### Participants and procedures

The population of the study consisted of healthy adolescents between the ages of 9–14 in Türkiye. The sample size of the study was calculated using the G*Power 3.1.9.7 analysis program; It was determined as 138 with 95% confidence interval, 5% margin of error, and 80% power. The research was conducted in primary and secondary schools with 151 randomly selected adolescents. Adolescents who were allowed to participate in the study by their families and volunteered themselves were included in the study. Adolescents who had communication disabilities (such as hearing problems), who could not communicate in Turkish, who had physical or mental chronic diseases, and who used medication for any reason were excluded from the scope of the research. The research was approved by the Bayburt University Research Ethics Committee (Decision no: 2023-42/5). All participants gave informed consent in accordance with the Declaration of Helsinki. In addition, all methods were carried out in accordance with the relevant guidelines of the journal. They are guaranteed the right to withdraw from the study at any time and the confidentiality of their personal information. All methods were conducted in accordance with relevant guidelines and regulations.

### Measures

The data were collected face-to-face with the Personal Information Form and Dysfunctional Attitudes Scale in Children (8–14 years) in an average of 15 min. Saliva samples were collected from children.

The children included in the study were told not to drink liquids, eat or brush their teeth 1 h before saliva intake, to stay away from tryptophan-rich dietary products such as red meat, eggs, fish, nuts, seeds and yoghurt, and not to brush their teeth. They were asked to keep a detailed food diary 24 h before saliva collection. Saliva samples were collected at a time period of 08:00–09:00 in the morning using the passive salivation method in Salivette tubes (Sarstedt, GERMANY) at a rate of 5 cc. After centrifuging at 2000 g for 20 min in a refrigerated centrifuge (NF 1200R, NUVE, Ankara, TÜRKİYE) in the laboratory, saliva samples were stored at -80 °C until analyzes for apelin hormone levels were performed. *Measurement of apelin hormone levels in salivary*: The minimum detectable concentration of the apelin hormone kit, which is used to measure apelin hormone levels in saliva samples obtained as a result of the study, has been reported as 7–1500 ng/L. The human-specific Human Apelin ELISA Kit (BT LAB, Cat.NoE2014Hu, CHINA) was used for the measurement of apelin level in the study. The determination of the ELISA hormone kit was studied in accordance with the procedure specified in the manufacturer’s catalog, using 3.47 ng/L, an intra-assay coefficient of 8.0%, and an inter-assay coefficient of 10.0%. The results were evaluated by reading absorption values at 450 nm in accordance with the procedure reported in the kit.

The personal information form was created by the researchers by examining the relevant literature. The form questioned the sociodemographic characteristics of the student, such as gender, age, income status, and parents’ age, educational status, and work status.

Dysfunctional Attitudes Scale in Children (8–14 Years): The Dysfunctional Attitudes Towards Children Scale, developed by D’Alessandro and Burton [6], consists of 22 items. This one-dimensional scale is designed as a five-point Likert type. Validity and reliability studies of the original scale were conducted on 453 students (221 girls, 232 boys) whose ages ranged from 8 to 14 and whose average age was 11.86. The American Psychiatric Association (APA) did not distinguish between children and adolescents in the depression diagnosis section of its diagnostic manual and listed the same basic symptoms. In addition, in this scale, which was prepared in line with the theoretical knowledge of Beck [3], who first discussed the concept of dysfunctional attitudes during his studies in the treatment of depression, no distinction was made between children and adolescents, and the 8–14 age group was chosen as the child age group. The name of the scale was determined as “Dysfunctional Attitudes Towards Children Scale”. The Cronbach Alpha internal consistency reliability coefficient of the scale was determined as 0.87, and the test-retest correlation was determined as 0.80. The unidimensional scale consisting of 22 items explains 27% of the total variance according to the exploratory factor analysis conducted within the scope of validity analysis. According to criterion-related validity studies, positive and significant relationships were determined between dysfunctional attitudes and depression (*r* =.30) and negative emotion (*r* =.29). High scores on the scale indicate that the individual has a high level of dysfunctional attitudes. The total score of individuals from the scale, which does not contain reverse-scored items, varies between 22 and 110 [6]. The validity and reliability study of the scale developed by D’Alessandro and Burton [6] was carried out by Oral and Günlü [22]. Scale, strongly disagree = 1 point; disagree = 2 points; neither agree nor disagree = 3; agree = 4; completely agree = 5 is scored with a 5-point Likert rating. As the child’s scores increase, the level of dysfunctional attitude increases. There are 22 items in the scale. A minimum of 22 points and a maximum of 110 points are obtained from the scale. There is no reverse coded item. The Cronbach Alpha reliability coefficient of the scale was determined as 0.82. In our study, the Cronbach Alpha reliability coefficient of the scale was found to be 0.85. Sample items in the scale: “I can only be happy if I know that everyone loves me.” “Young people should be the best at everything they do.” “People should be upset if they can’t have what they want.” “Whenever I make a mistake, bad things always happen.” “I have to be better than other kids.” “If I fail once, I always fail.” “I have to be angry with myself when I make a mistake.” “If I need help, it means I am inadequate.”

### Data Analysis

Data were evaluated using the Statistical Package for the Social Sciences (SPSS) 26.0 for Windows (SPSS, Chicago, Il, USA) package program. Whether the data were normally distributed or not was evaluated by the Skewness and Kurtosis coefficients being in the range of (-1)–(+ 1) [23]. Number, percentage, mean (X̄) and standard deviation (SD) values for continuous variables were used for descriptive statistics. Parametric tests were applied because the data were normally distributed. The Independent Samples Test and One-Way ANOVA test were used to compare the descriptive characteristics of the children with their scale scores, salivary apelin levels. Bonferroni posthoc analysis was used in groups with homogeneous distribution, to determine which group caused the difference in more than two groups with equal group variances. Whether there was a relationship between the variables was evaluated with Pearson correlation analysis. *p* <.01 and *p* <.05 significance levels were used as statistical significance values.

## Results

Table 1 includes the comparison of the sociodemographic characteristics of the adolescents, their salivary apelin hormone levels and the scale total score averages. The average age of the adolescents participating in the study was 11.52 ± 1.69, 45.7% were girls and 54.3% were boys. 38.4% of the mothers are primary school graduates, 84.8% are housewives; 41.7% of the fathers are high school graduates and 32.5% are retired/other. 82.8% of the adolescents perceived their income status as medium. There was no significant difference between the adolescents’ average salivary apelin hormone levels and dysfunctional attitudes scale scores and the mother’s education level, mother’s profession, father’s education level, father’s profession and income (*p* >.05).

**Table 1:** Comparison of sociodemographic characteristics of children with Apelin hormone levels and scale total score (*N* = 151)

Variables	N (%)	Apelin hormone levels	Scale total score
Gender			
Girl	69(45.7)	0.696 ± 0.052	52.95 ± 14.43
Boy	82(54.3)	0.671 ± 0.047	59.04 ± 14.22
		**t = 3.101**	**t=-2.790**
		***p*** **=** .002*	***p*** **=** .006*
Age			
9-10^1^	50(33.1)	0.647 ± 0.044	67.52 ± 12.25
11–12^2^	49(32.5)	0.690 ± 0.034	54.87 ± 8.46
13–14^3^	52(34.4)	0.710 ± 0.051	46.75 ± 10.92
		**F = 26.809** ^**a**^	**F = 48.814** ^**b**^
		***p*** **=** .000**	***p*** **=** .000**
Perception of income status			
Low	11(7.3)	0.689 ± 0.057	56.63 ± 15.44
Medium	125(82.8)	0.683 ± 0.051	55.45 ± 13.55
High	15(9.9)	0.674 ± 0.039	62.73 ± 12.37
		F = 0.310	F = 1.927
		*p* =.734	*p* =.149
Mother’s education level			
Primary school	58(38.4)	0.691 ± 0.048	55.12 ± 13.34
Middle school	40(26.5)	0.678 ± 0.054	57.75 ± 14.57
High school	42(27.8)	0.675 ± 0.052	56.59 ± 13.74
University and above	11(7.3)	0.684 ± 0.047 F = 0.891	55.63 ± 12.88 F = 0.305
		*p* =.448	*p* =.822
Mother’s occupation			
Housewife	128(84.8)	0.684 ± 0.052	56.77 ± 13.98
Civil servant/worker	13(8.6)	0.675 ± 0.038	56.23 ± 8.63
Retired/other	10(6.6)	0.675 ± 0.056	49.80 ± 14.21
		F = 0.300	F = 1.211
		*p* =.742	*p* =.301
Father’s education level			
Primary school	30(19.9)	0.688 ± 0.040	54.40 ± 12.77
Middle school	32(21.2)	0.666 ± 0.056	61.43 ± 14.91
High school	63(41.7)	0.691 ± 0.050	54.61 ± 12.53
University and above	26(17.2)	0.675 ± 0.053 F = 2.098	56.03 ± 14.84 F = 2.064
		*p* =.103	*p* =.108
Father’s occupation			
Tradesman	26(17.2)	0.680 ± 0.041	57.11 ± 10.86
Civil Servant	32(21.2)	0.684 ± 0.047	54.03 ± 12.78
Laborer	44(29.1)	0.675 ± 0.049	59.02 ± 13.31
Retired/other	49(32.5)	0.689 ± 0.059 F = 0.600	54.79 ± 15.64 F = 1.107
		*p* =.616	*p* =.348

*Independent T test **One-Way ANOVA ^a^Bonferroni= 3 > 1, 2 > 1 ^b^Bonferroni= 1 > 2, 1 > 3, 2 > 3

In the study, the mean level of salivary apelin hormone was found to be 0.696 (SD 0.052) ng/mL in the female gender, while it was 0.671 (SD 0.047) ng/mL in the male gender. According to the age of the adolescents, mean salivary levels of apelin hormone were determined as 0.647 (SD 0.044) pg/mL, 0.690 (SD 0.034) ng/mL and 0.710 (SD 0.051) ng/mL for 9–10, 11–12 and 13–14 year olds, respectively. A significant difference was found between the mean level of salivary apelin hormone and the gender and age variables of adolescents (*p* <.01). Apelin hormone levels of female adolescents were higher than males. As a result of the posthoc analysis made in terms of age variable, the salivary apelin hormone level was found to be between 13 and 14 and 11–12 years old; It was determined that those aged 11–12 years were higher than those aged 9–10. In the study, the mean scores of the Dysfunctional Attitudes Scale (DAS) of adolescents were found to be 52.95 (SD 14.43) in females and 59.04 (SD 14.22) in male adolescents. A significant difference was found between the total mean scores of DAS and the gender variable of the adolescents (*p* <.01). The mean total score of DAS in male adolescents was found to be higher than in female adolescents. A significant difference was found between the mean DAS total score and the age variable of the adolescents (*p* <.01). As a result of the posthoc analysis, the total score averages of the DAS were found to be between 9 and 10 years old and 11–12 and 13–14 years old; It was determined that those aged 11–12 were higher than those aged 13–14 (Table 1).

In comparisons made for the average salivary apelin level and dysfunctional attitudes of the adolescents in each age group according to the gender variable; It was determined that the apelin levels of the 13–14 age group girls were the highest and the dysfunctional attitude levels were the lowest (*p* <.05) (Table 2).

**Table 2:** Comparison of salivary apelin hormone levels and scale total score according to gender variable for age groups (*N* = 151)

Age	Gender	N (%)	Apelin hormone levels	Scale total score
9–10	Girl	17(34.0)	0.651 ± 0.045	66.58 ± 10.88
	Boy	33(66.0)	0.645 ± 0.044	68.00 ± 13.03
			t = 0.492	t=-0.383
			*p* =.625	*p* =.704
11–12	Girl	22(44.9)	0.695 ± 0.029	54.72 ± 7.55
	Boy	27(55.1)	0.685 ± 0.037	55.00 ± 9.28
			t = 1.004	t=-0.111
			*p* =.321	*p* =.912
13–14	Girl	30(57.7)	0.722 ± 0.053	43.93 ± 11.78
	Boy	22(42.3)	0.693 ± 0.043	50.59 ± 8.43
			**t** **=** **2.126**	**t=-2.257**
			***p*** **=** .038*	***p*** **=** .028*

*Independent T test

Table 3 shows the relationship between adolescents’ dysfunctional attitudes and average level of salivary apelin hormone. A negative, strong and statistically significant correlation was found between the dysfunctional attitudes of adolescents and the mean level of salivary apelin hormone (*p* < 0.01) (Table 3).

**Table 3:** The relationship between children’s apelin hormone levels and scale total score (*N* = 151)

Variables		Apelin hormone levels	Scale total score
**Apelin hormone levels**	r	1	
	p		
**Scale total score**	r	− 0.778^**^	1
	p	0.000	

**Correlation is significant at the 0.01 level (2-tailed)

## Discussion

The foundations of dysfunctional attitudes are laid in childhood and continue to develop throughout life along with cognitive development from childhood. These are very strong attitudes and can have a direct or indirect effect on the development of many health problems. Studies on this subject are mostly based on the adult age group. However, it is important to evaluate dysfunctional attitudes in children, whose foundations are laid in childhood and can lead to many negativities.

Apelin is an adipokine that has different functions in many physiological systems and many metabolic and physiological processes, including fluid electrolyte and energy balance, insulin, glucose, lipid metabolism, as well as the cardiovascular, immune and reproductive systems [24, 25]. Apelin hormone is reported to have a neuroprotective role in neurological diseases as well as controlling emotional behavior depending on age and metabolic status [26, 27]. In addition, it has been reported that the hormone apelin is an important determinant of plasma apelin levels of puberty [28]. Although the mean salivary apelin hormone levels of the adolescents included in the study increased with age, it was determined that the mean salivary apelin level in female adolescents was higher than in male adolescents in terms of gender (*p* <.05). The results of our current study are the first to examine the effect of apelin on hormone levels in terms of dysfunctional attitudes, age and gender. Although the studies are limited, they are similar to the results of similar studies reported in the literatüre [25, 29].

In the study, we found a significant difference between the mean scores of the DAS and the age variable of the adolescents (*p* <.01). In the study, it was found that the DAS total score average was higher in young adolescents than in others. Contrary to our study finding, Pesen and Çelik [27] reported in their study with university students that there was no significant difference between the total score averages of the dysfunctional attitudes scale used for adults and the age variable. The reason for this is thought to be due to the different characteristics of the sample groups included in the studies. The foundations of dysfunctional attitudes are laid in childhood and continue their lifelong development along with cognitive development from childhood [5, 30]. Beck [7] states that the factors affecting dysfunctional attitudes that begin to take shape in early childhood are the education provided by the parents in early childhood, taking the parents as an example and experiences, and the traumas experienced by the children. For this reason, it can be said that dysfunctional attitudes are more affected by the beliefs, culture and values of the environment in which the person lives rather than the environment in which he grows up [31].

In our study, the DAS total mean score of male adolescents was found to be higher than that of female adolescents. Similar to our study finding, in the study of Pesen and Çelik [26] the dysfunctional attitude scores of male university students were found to be higher than that of females. On the other hand, Çivitçi [32] and Kılıç [33] reported that female students have higher dysfunctional attitudes than male students in their studies. This finding suggests that girls may be more sensitive and perfectionist [33]. There are also studies in the literature that indicate that dysfunctional attitudes do not differ depending on gender [34, 35]. The inconsistency of the results of the studies in the literature suggests that it may be due to the differences in the sociocultural and sociodemographic characteristics of the sample groups.

This study had some limitations. Participants of the study were limited to children aged 9–14 years. Additionally, evaluating only sociodemographic variables is another limitation of the study. Moreover, and more importantly, variables such as previous traumas experienced by adolescents, learning factors, parental styles, biological factors, and social context, which may affect the results of the study, should be taken into account when evaluating the results, as they cannot be excluded. The application of the research with different variables in different age groups will contribute to the literature. Despite these limitations, the study also had its strengths. This study is valuable because it is the first study to examine salivary apelin levels and dysfunctional attitudes together in adolescents.

As a result, there is a negative, strong and statistically significant relationship between the salivary apelin hormone levels and dysfunctional attitudes of adolescents (*p* < 0.01). Dysfunctional attitudes are negative beliefs that individuals form as a result of communication with other people and against oneself, others and the world, and are a condition seen in individuals experiencing depression. Apelin hormone, which is reported to be a biomarker that controls emotional behavior depending on age and metabolic status, has emotional behavior regulatory and antidepressant functions. Considering the consequences of dysfunctional attitudes on depression and negative mental health in children, which can be neglected, nurses working in the field of pediatrics have important roles. Nurses can be effective in early diagnosis by identifying risk groups. Nursing practices are of great importance in protecting, developing and caring for the health of adolescents.

## Data Availability

No datasets were generated or analysed during the current study.
