# Correction: salivary apelin hormone response and dysfunctional attitudes in adolescents in Türkiye: a relational screening model

**DOI:** 10.1186/s40359-024-01728-3

**Published:** 2024-04-24

**Authors:** Zila Özlem Kirbaş, Bülent Bayraktar, Elif Odabaşi Aktaş

**Affiliations:** 1 Faculty of Health Sciences, Department of Nursing, Bayburt University, Bayburt, Türkiye; 2 Faculty of Health Sciences, Department of Physiotherapy and Rehabilitation, Bayburt University, Bayburt, Türkiye; 3 Faculty of Health Sciences, Department of Midwifery Bayburt, Bayburt University, Bayburt, Türkiye

**Correction to: BMC Psychology (2024) 12:64**

10.1186/s40359-024-01551-w

Following publication of the original article, it came to the journal’s attention that the order of the citations (following citation of reference 22) was incorrect. The order has since been corrected in the published article.

